# Depression moderates the association between pregnancy and suicidal ideation among pregnant and non-pregnant reproductive age women in Brazil

**DOI:** 10.3389/fpsyt.2022.1029048

**Published:** 2022-11-28

**Authors:** Alexandre Faisal-Cury, Karen M. Tabb, Jessica Mayumi Maruyama, Alicia Matijasevich

**Affiliations:** ^1^Departamento de Medicina Preventiva da Faculdade de Medicina FMUSP, Universidade de São Paulo, São Paulo, Brazil; ^2^School of Social Work at the University of Illinois at Urbana-Champaign, Urbana, IL, United States

**Keywords:** depression, pregnancy, suicidal ideation, Brazil, gravid

## Abstract

**Background:**

Maternal mental health problems are a serious public health concern. Previous data reported that pregnancy might have a protective effect against suicide. In contrast, more recent studies suggested that the prevalence of suicidal ideation (SI) is higher among pregnant women compared to the general population. Using a nationally representative population-based sample of Brazilian reproductive-aged women, this study aims to assess whether SI is more prevalent among pregnant women in comparison with nonpregnant woman.

**Methods:**

We used data from the Brazilian National Health Survey (PNS) of 2019, a cross-sectional study that comprised a representative sample of residents in private households in Brazil. For the analysis of this study, we selected women aged between 15 and 49 years old who have answered the questionnaire of the Selected Resident of the PNS, which comprised a sample of 27,249 women. Logistic regression models were performed to obtain crude and adjusted odds ratios (OR) and 95% confidence intervals (95%CI) for the association between pregnancy status and SI.

**Results:**

The prevalence of SI during pregnancy was 6.8% (95% CI: 6.2–7.4). The association between pregnancy status and SI was modified according to the recent clinical diagnosis of depression (interaction term: OR = 41.72, 95% CI: 5.64–308.45, *p* < 0.001). Our findings indicated that among nondepressed women, pregnancy status seems to decrease the probability of SI. Additionally, SI is associated with a vulnerable profile that includes being an adolescent, having an unpartnered/not married status, lower family income, lower education, and a recent clinical diagnosis of depression.

**Conclusion:**

SI is a common problem for reproductive-age women. In the presence of a recent depression clinical diagnosis, pregnancy increases the risk of SI. Management of SI among pregnant women should correctly identify sociodemographic risk factors and the presence of a recent clinical diagnosis of depression.

## Introduction

Nearly 800,000 people die due to suicide every year worldwide, which is one person every 40 s. Suicide is a global phenomenon, accounting for 1.4% of all deaths worldwide. Nevertheless, 79% of suicides occurred in low- and middle-income countries in 2016 (1). A meta-analysis found a suicide pooled prevalence of 1.00% (95% CI: 0.54–1.57) (3). Suicide is also a leading cause of death during the perinatal period in high-income countries (ac-counting for 5% to 20% of maternal deaths) (2) and has a modest contribution in low-middle income countries. Previous data has reported that pregnancy might have a protective effect against suicide (6–8). In contrast, more recent studies have suggested that the prevalence of suicidal ideation is higher among pregnant women (9, 10).

Suicidal ideation (SI) is considered an important precursor of later suicidal death (11, 12). Gender differences exists where SI and depression are 2–3 times higher in women in comparison with men (4, 5). Identification of women who present SI may be a great opportunity for the use of suicide prevention measures. Several risk factors are associated with SI including demographic characteristics, markers of socioeconomic status, intimate partner violence, and comorbid psychiatric conditions. Nevertheless, there is conflicting evidence whether pregnant women are at higher risk of endorsing SI in comparison with reproductive-aged non-pregnant women.

Overall, the SI prevalence during pregnancy varies widely across studies, showing a range from 3 to 33% (10). The highest estimates of antenatal SI (23–33%) have been found from studies performed in the US (9, 13). Difficulties in assessing antenatal SI include the lack of a proper and valid screening instrument, stigmatization of suicide, and time constraints in prenatal care clinics (14). Frequently, SI is assessed along with depressive symptoms screening rather than separately. This screening strategy is largely explained by the strong association between SI and antenatal depressive symptoms reported in several studies (12, 15). Nevertheless, studies also have shown that many women without a depression diagnosis have suicidal thoughts. Data from the World Mental Health Surveys (16) reveal that only 16.6% of people with suicidal ideation have diagnosable depression.

In Brazil, there are a few studies about SI and its associated factors among pregnant and non-pregnant women. The estimates of SI prevalence during the antenatal period among studies varied between 6.2 (20) and 8.1% (21). Two previous population-based studies assessed SI prevalence. In one study, Carpena and colleagues found a 4.9% SI prevalence among women in the 2013 Brazilian National Health Survey (*N* = 34,282 women) (22). In a second study, while data from the Brazilian Longitudinal Study of Adult Health showed that among Brazilian middle-aged women the prevalence of the symptoms of “tiredness of life” and “suicidal thoughts” were, respectively, 4.3 and 0.9% in the 7 days prior to the survey (23). Overall, there is a lack of population-based studies in Brazil comparing SI prevalence between pregnant and non-pregnant women.

Accordingly, in this study we aimed to assess whether SI is more prevalent among pregnant women in comparison with non-pregnant woman using a nationally representative population-based sample of Brazilian reproductive-aged women. In addition, we aimed to evaluate if a recent clinical diagnosis of depression (less than 1 year prior to the survey) would moderate the association between pregnancy and suicidal ideation.

## Materials and methods

### Design and sample

This study is based on the 2019 Brazilian National Health Survey (PNS). This study uses a subsample of the Master Sample of the Instituto Brasileiro de Geografia e Estatística's (IBGE's) Integrated Household Survey System for the PNS. A cross-sectional study, the PNS is drawn from a representative sample of residents in private households in Brazil. The sample is large enough to enable the estimation of precise prevalence rates and 95% confidence intervals, taking the clustered sample in multiple stages into account, for chronic conditions such as diabetes, hypertension, and depression; experiencing violence; tobacco use disorder and alcohol consumption; use of health services and having health insurance; and practice of physical exercise. The master sample from which it the PNS drawn omits the census areas with very small population as well as barracks and residential institutions, but otherwise represents the Geographic Operational Base of the Demographic Census 2010. The Survey Report (24) offers more details on PNS methods. For the analysis of this study, we selected women aged between 18 and 49 years old who had answered the questionnaire of the Selected Resident of the PNS, therefore comprising a sample of 27,249 women. The PNS 2019 response rate was 91.9%.

### Main outcome variable

The last question on the PHQ-9 is as follows: “*In the last two weeks, how many days did you think about hurting yourself in any way or think that it would be better to be dead?*” The main outcome, suicidal ideation, was considered positive for participants who answered anything other than “*None*,” whether it was “*Almost every day*,” “*Once a week or more*,” or “*Less than once a week*.”

### Recent clinical diagnosis of depression

The 2019 PNS does not use structured interviews for the assessment of recent clinical diagnosis of depression. Rather, clinical diagnoses is captured from two questions: first “*Has a medical doctor or other mental health professional (such as a psychiatrist or psychologist) ever diagnosed you with depression*?” and the second question was “*What was your age when you received a depression diagnosis*?” Participants who indicated they had never been diagnosed with depression or that they had been diagnosed more than a year prior to the survey were considered not to have a recent clinical diagnosis of depression; anything less than 12 months prior to the survey was categorized as recent.

### Main exposure variable

Participants were asked, “*Are you currently pregnant*?” Women who indicated “*I don't know*” (*N* = 113) were omitted from the sample. Those who answered “Yes” or “No” were classified according to their response.

### Covariates

Age was categorized as 15–19, 20–34, and 35–49 years. Years of schooling were also categorized: 0–8, 9–11, >11 and adjusted by age. Family monthly income was assessed in quartiles: 0–304 USD, 305–560 USD, 561–1044 USD, and ≥1045, with 1 USD **=** 3.83 Brazilian reais. We calculated household income per capita by dividing reported monthly income by the reported number of people in the household. Having a private medical plan and living with a partner were both yes/no variables. Skin color and marital status were also binary, White/Non-white, Married/Other. Residence was measured with two binary variables: Urban/ Rural and South-Southeast/North-Northeast-Midwest.

### Statistical analysis

To undertake the descriptive analysis, first, all variables were categorized. Logistic regression models offered crude and adjusted odds ratios (OR) and 95% confidence intervals (95% CI) of the association between SI and pregnancy status. Age, skin color, whether they were living with a partner, years of schooling, whether they have a private medical plan, the residential variables, and household monthly income per capita were all confounding variables. Including an interaction term in the fully adjusted model revealed that recent clinical diagnosis of depression had a moderating effect. To assess the moderating effect of recent clinical diagnosis of depression, we included covariates with a *p* < 0.2 in the bivariate analysis in the fully adjusted model. We considered a *p* < 0.05 as statistically significant for the interaction term. We estimated predicted probabilities of suicidal ideation using the predictive margins command in STATA (25). Statistical analysis was performed using STATA 16 software. All analyses were performed considering the weighting for the complex sample structure, in order to represent the Brazilian population, according to the research sample (*svy* command in STATA).

### Ethical aspects

The National Research Ethics Committee (Conselho Nacional de Ética em Pesquisa) of the National Health Council (Conselho Nacional de Saúde; number 328.159) approved the PNS study in June 2013. Participants could answer the questionnaire could be answered in whole or in part and participations was voluntary. The IBGE website provides the PNS dataset with identifying information omitted.

## Results

**Figure 1:**
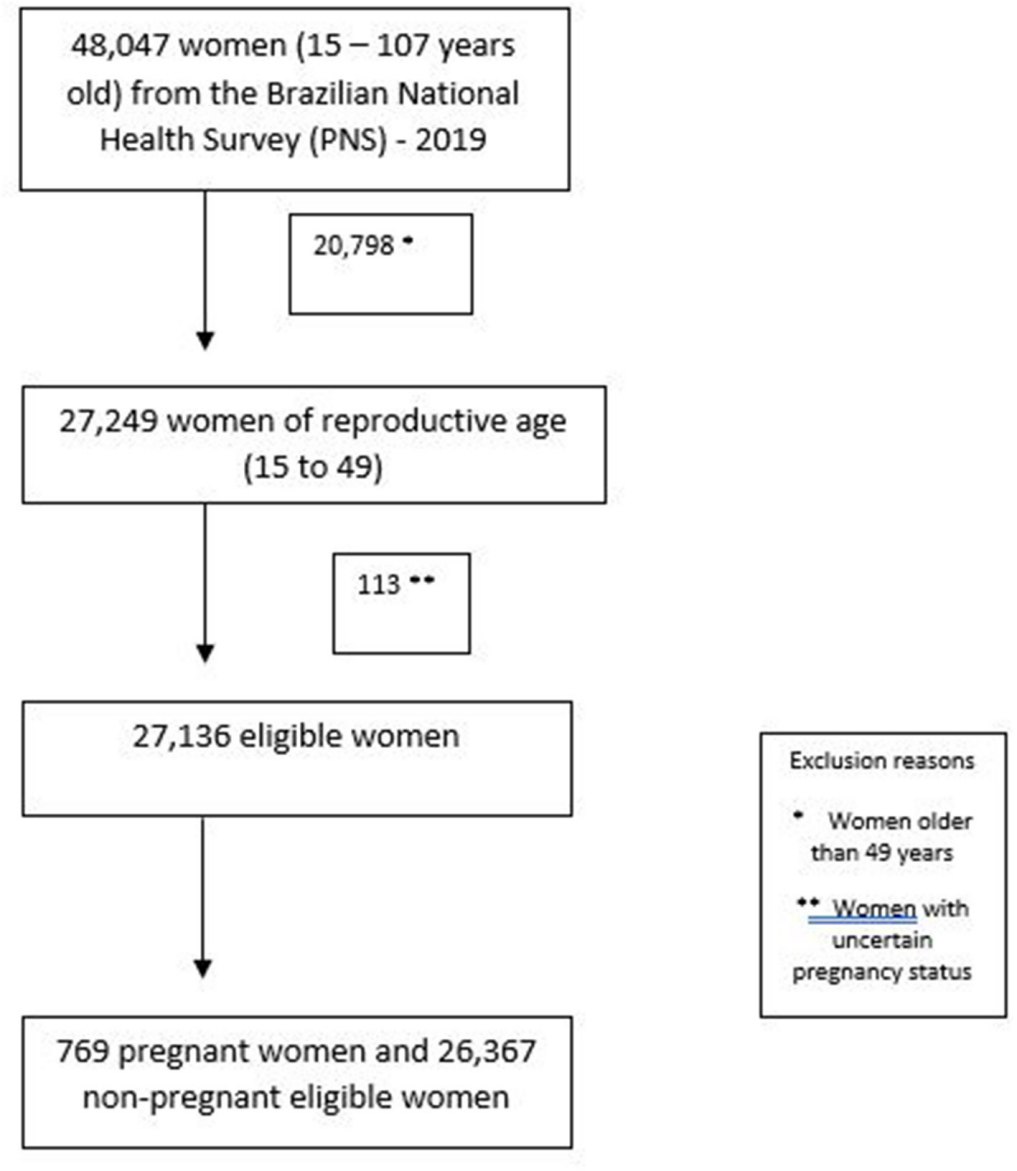
Participants flowchart. ^*^Women older than 49 years. ^**^Women with uncertain pregnancy status.

Of the 48,047 women (15–105 years old) in the PNS 2013 dataset, there were 27,249 women of reproductive age (15 to 49 years old) with information about pregnancy status. Of those women, 113 were not sure about being pregnant and were excluded from the analysis, leaving a total of 27,136 participants representing a women's population size of 69,682,429. Our final sample included 26,367 non-pregnant women and 769 pregnant women (Figure 1). The main characteristics of our sample were 41.0% were white, 58.4% had a partner, and 56.5% were between 35 and 49 years old (mean age 34.8, range 15–49). In relation to socioeconomic status, 30.7% had less than 8 years of schooling and 16.7% had a family monthly income of less than 304 USD. The majority of women (73.5%) did not have private health insurance. The greater part lived in the south/southeast region of the country (55.6%) and in an urban area (87.4%). Regarding mental health characteristics, only 1% (95% CI 08:1.2) had had depression for less than 1 year (Table 1) and 12.9 % (95% CI 12.0:13.6) had had a depression diagnosis for more than 1 year. The overall prevalence of SI was 6.8% (95% CI 6.2:7.4) ranging from 6.5% (95% CI 5.9:7.0) among women without recent depression to 36.0% (95% CI 25.1:48.6) among women with recent depression.

**Table 1:** Women characteristics according to suicidal ideation in the National Health Survey, Brazil 2019.

**Variables**	**Prevalence (%)**	**95%CI**	**Suicidal ideation % (95% CI)**	**P level**
**Pregnant status**				0.09
No	97.6	97.3–97.9	6.8 (6.3–7.4)	
Yes	2.4	2.0–2.6	4.4 (2.6–7.2)	
**Age (years)**				< 0.001
35/49	56.5	55.9–56.9	6.4 (5.7–7.2)	
20/34	32.4	31.7–33.0	5.8 (5.1–6.6)	
15/19	11.1	10.7–11.5	11.4 (9.2–14.0)	
**Race/color**				< 0.001
White	41	39.8–42.1	5.2 (4.5–6.1)	
Non-white	59	57.8–60.1	7.8 (7.1–8.6)	
**Years of Schooling**				< 0.001
> 11	24.6	23.6–25.5	3.3 (2.7– 4.0)	
11-Sep	44.6	43.5–45.7	6.7 (5.8–7.6)	
0–8	30.7	29.7–31.8	9.6 (8.4–10.9)	
**Monthly income (USD)**				< 0.001
1.045/max	32.6	31.3–33.8	4.8 (3.9–5.9)	
561–1.044	29.3	28.2–30.3	6.1 (5.1–7.4)	
305–560	21.4	20.5–22.2	7.9 (6.9–9.1)	
0–304	16.7	16.0–17.3	10.1 (8.9–11.4)	
**Live with partner**				< 0.001
Yes	58.4	57.5–59.3	5.7 (5.0–6.5)	
No	41.6	40.6–42.4	8.2 (7.3–9.2)	
**Marriage status**				< 0.001
Married	37.8	36.8–38.7	5.1 (4.2–6.2)	
Other	62.2	61.2–63.1	7.7 (7.0–8.5)	
**Urban Area**				0.53
Yes	87.4	86.8–87.9	6.7 (6.1–7.3)	
No (rural)	12.6	12.1–13.1	7.1 (6.0–8.4)	
**Country region**				0.31
South/Southeast	55.6	54.5–56.6	6.5 (5.7–7.4)	
Other	44.4	43.3–45.4	7.1 (6.5–7.7)	
**Private health insurance**				< 0.001
Yes	26.5	25.3–27.6	4.1 (3.3–5.0)	
No	73.5	72.3–74.6	7.7 (7.0–8.4)	
**Depression less than 1 year**				< 0.001
No	98.9	98.7–99.1	6.5 (5.9–7.0)	
Yes	1.01	0.8–1.2	36.0 (25.1–48.6)	

The higher prevalence of SI was found among participants who were 15 to 19 years of age (11.4%), with non-white skin color (7.8%), with the lowest years of education (9.6%), with the lowest family monthly income (10.1%), without a partner (8.2%), without a private health plan (7.7%) and who were not pregnant (6.8%). The highest SI prevalence was seen among women who had reported depression for less than 1 year (36.0%; Table 1).

In the binomial logistic regression, the following variables were associated with an increased risk of SI: 15 to 19 years of age (OR: 1.87; 95% CI 1.41:2.47), non-white skin color (OR: 1.53; 95% CI 1.26:1.85), with 9–11 years of education (OR: 2.07; 95% CI 1.61: 2.65); with 0–8 years of education (OR: 3.08; 95% CI 2.40: 3.95), with a family monthly income of 305–560 USD (OR: 1.69; 95% CI 1.30:2.20), with a family monthly income of 0–304 USD (OR: 2.21; 95% CI 1.71:2.85), without a partner (OR: 1.47; 95% CI 1.22:1.76), not married (OR: 1.54; 95% CI 1.22:1.93), and without a private health plan (OR: 1.93; 95% CI 1.52:2.45). In relation to clinical diagnosis of depression, depression for less than 1 year was associated with an increased risk of SI (OR: 8.12; 95% CI 4.8:13.7; Table 2).

**Table 2:** The odds ratio (OR) and 95% confidence intervals (CI) of suicidal ideation according to sociodemographic and obstetric risk factors in the National Health Survey, Brazil 2019.

**Variables**	**Suicidal ideation**	
	**Crude**	**P level**
	**OR (95%CI)**	
**Pregnancy status**		0.09
No	1	
Yes	0.63 (0.37–1.07)	
**Age (years)**		0.002
35/49	1	
20/34	0.90 (0.75–1.08)	
15/19	1.87 (1.42–2.47)	
**Race/color**		< 0.001
White	1	
Other	1.53 (1.26–1.85)	
**Years of Schooling**		< 0.001
11	1	
9-11	2.07 (1.61–2.65)	
0–8	3.08 (2.40–3.95)	
**Monthly income (USD)**		< 0.001
1.045/max	1	
561–1.044	1.28 (0.95–1.74)	
305–560	1.69 (1.30–2.20)	
0–304	2.21 (1.71–2.85)	
**Live with partner**		< 0.001
Yes	1	
No	1.47 (1.22–1.76)	
**Marriage status**		< 0.001
Married	1	
Other	1.54 (1.22–1.93)	
**Urban Area**		0.53
Yes	1	
No (rural)	1.06 (0.87–1.30)	
**Country region**		0.31
South/Southeast	1	
Other	1.09 (0.92–1.29)	
**Private health insurance**		< 0.001
Yes	1	
No	1.93 (1.52–2.45)	
**Depression less than 1 year**		< 0.001
No	1	
Yes	8.12 (4.8–13.7)	

**Figure 2:**
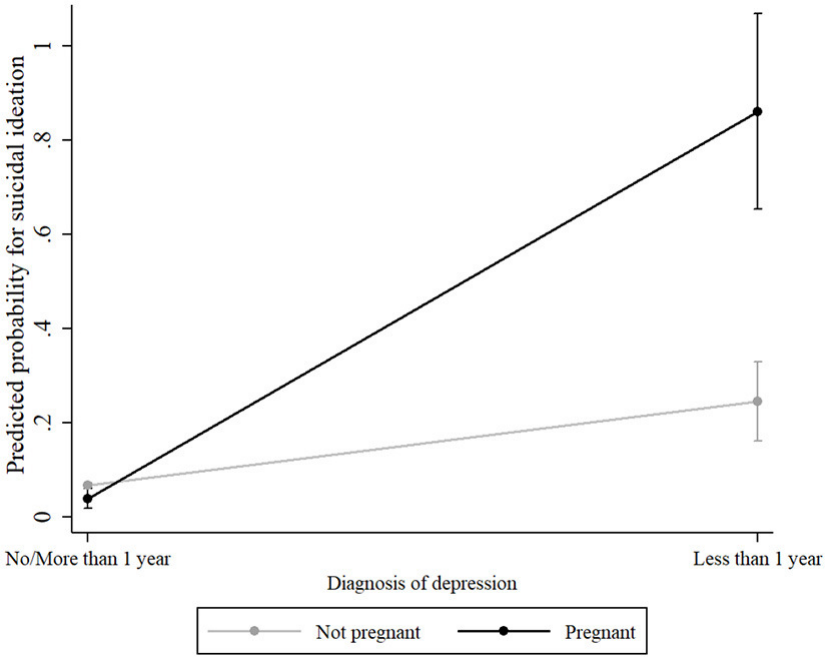
Predictive probability for suicidal ideation by pregnancy status and diagnosis of depression. Figure 2 was drawn by estimating the predictive probability of the interaction effect of pregnancy status x diagnosis of depression and plotting them with 95% CI by predictive margins and marginsplot of STATA.

The association between pregnancy status and suicidal ideation was modified according to the recent clinical diagnosis of depression (interaction term: OR = 41.721, 95% CI: 5.643–308.451, *p* < 0.001). Table 3 and Figure 2 show that among non-depressed participants, pregnant women showed a slightly lower probability of presenting suicidal ideation (predicted probability = 0.039, 95% CI 0.017; 0.060) when compared to non-pregnant women (predicted probability = 0.067, 95% CI 0.061; 0.072). Conversely, pregnant women who were recently diagnosed as depressed presented a probability of 0.861 (95% CI 0.653; 1.067) for suicidal ideation, in contrast to a 0.245 (95% CI 0.160; 0.329) probability among non-pregnant women with a recent diagnosis of depression.

**Table 3:** Predictive probability of suicidal ideation by pregnancy status and diagnosis of depression.

	**Diagnosis of depression**
	**No/More than 1 year**	**Less than 1 year**
**Suicidal Ideation**	**Predicted probability**	**Predicted probability**
	**(95% CI)**	**(95%CI)**
No	0.067 (0.061–0.072)^*^	0.245 (0.160–0.329)^*^
Yes	0.039 (0.017–0.060)^*^	0.861 (0.653–1.067)^*^

* *p* < 0.001. Interaction term: OR = 41.721 (95% CI: 5.643 – 308.451), *p* < 0.001.

In other words, pregnant women without recent depression (< 1 year) are less likely to report SI than non-pregnant women without recent depression (< 1 year). On the other hand, the probability of SI in pregnant women with recent depression is approximately 90% while in non-pregnant women with recent depression, this probability is approximately 25%.

## Discussion

In the present study, we showed that SI is frequent (6.8%) and it is associated with a vulnerable profile that includes being an adolescent, having an unpartnered/not married status, lower family income and education, and a recent clinical diagnosis of depression. SI was more prevalent among non-pregnant women (6.8%) in comparison with pregnant women (4.4%), although it was not statistically significant. Nevertheless, the relationship between pregnancy status and SI was moderated by the length of depression diagnosis.

Our findings indicate that among non-depressed women, pregnancy status seems to decrease the probability of suicidal ideation. However, a recent clinical diagnosis of depression substantially increases the probability of suicidal ideation in both pregnant and non-pregnant women, with pregnant women being largely vulnerable to suicidal ideation in the presence of depression. Although the focus of our study is not the assessment of factors associated with SI, we found that several variables were associated with SI including lower family income, lower years of schooling, and younger mother's age. The proportion of pregnant adolescents with SI (11.4%) was much higher than in other age groups (approximately 6%). This group of women deserves special attention in a national context where guarantees of reproductive rights are lacking (26).

These results may help to explain the lack of agreement between studies regarding the role of pregnancy as a risk factor for SI. Previous studies reporting a protective effect of pregnancy employed different study designs and methods of assessment: analysis of hospital discharge data files (27), case control study (7), enquiries into maternal deaths (28), and autopsy reports (29). Despite this protective effect subgroups of women with mental disorders may show an elevated risk of suicide during pregnancy and following birth (6). A review that evaluated prevalence estimates of suicidality (suicide deaths, attempts, and ideation including thoughts of self-harm) in pregnancy and the postpartum period found that relative to suicide rates in the general female population, pregnant women have lower suicide rates. According to these authors this data cannot be explained solely by depression severity (12). Increased social support, concern for the unborn child, and more contact with health care providers may work together to reduce the risk of suicide (12).

On the other hand, more recent cohort studies have reported that pregnancy is associated with an increased risk of SI. Kim et al. used the Edinburgh Postnatal Depression Scale (EPDS) in 22,118 perinatal women to report an SI prevalence of 4.1 and 3.4% during pregnancy and at 6 weeks after delivery, respectively (30). A cohort study in Japan used the EPDS to assess 430 women and found that SI was higher in late pregnancy (5.8%) in comparison with 1 month after delivery (31). Unfortunately, both studies evaluated only perinatal women, which limits further comparisons with non-pregnant reproductive aged women. Gelaye et al. (32) reviewed SI prevalence from studies performed in several countries and reported that SI may be more common among pregnant women than the general population. Nevertheless, this conclusion was based on an indirect comparison of results from different studies (32).

In contrast with studies reporting either an increased or a decreased risk of SI during pregnancy, a different picture emerged from a large community-based study with a diverse sample of pregnant women (*N* = 2,159). The authors used item 9 from the PHQ-9 to find an SI of 2.7%, which was similar to rates reported in nationally representative samples of non-pregnant women (15). Zhong et al. (17) evaluated 1,517 pregnant women attending prenatal care clinics in Lima, Peru, and found that 49% of participants who screened positive for suicidal ideation had negative results in the depression screening (Patient Health Questionnaire-9 [PHQ-9] score ≤ 10). Even participants with low PHQ-9 scores (e.g., 3, 4, and 5) still endorsed suicidal ideation (i.e., responded positively to item 9 of the PHQ-9) (17). One possible limitation with this approach is the fact that depressive symptoms may be an effect modifier of the relationship between pregnancy and SI. In other words, in the presence of depression, the pregnancy could act as a further stressor (or a buffering factor) for the development of suicidal thoughts. Therefore, a higher risk of SI among pregnant women may vary not only according to sociodemographic and interpersonal factors but also with a pregnant woman's previous history of depression. For example, Garman et al. (18) suggested that among perinatal women, depression and suicide are overlapping but independent phenomena, especially among older and more chronically depressed women (18). Additionally, women who scored low on depressive symptoms and women with non-specific, but clinically relevant, symptoms of anxiety and depression (e.g., insomnia, headache, fatigue, irritability) that do not meet diagnostic criteria for particular Axis I disorders deserve attention since they can also show SI (19).

Despite this conflicting evidence, most of the studies agreed that SI is highly associated with depression (and psychiatric disorders) and managed this variable in their analyses. This is advisable considering that up to half of pregnant women referred for psychiatric care already have a history of self-harm (33). Nevertheless, a distinctive feature of our study in comparison with other studies is the analysis of a moderating effect of depression in the relationship between pregnancy and SI. To the best of our knowledge, no other study has employed the same type of analysis.

Our results, derived from a nationally representative sample of reproductive age, may have public health implications. SI affects 6.8% of women and during pregnancy has serious fatal and non-fatal adverse outcomes for both mother and infant (7, 9, 15, 34, 35). Fortunately, there is also evidence that suicidal thoughts may subside alongside depressive symptoms throughout pregnancy (18). Nevertheless, despite the lack of a specific screening instrument, SI should be assessed during pregnancy, especially among women with a recent diagnosis of depression. New SI screening strategies have been recently developed with good results (36) and should be replicated in future studies. Meanwhile, among newly depressed women, pregnancy offers a clear opportunity for suicide risk reduction and prevention.

### Strengths and limitations

The present study has the following strengths. Our analysis was based on data from the PNS 2019, a large population-based survey with a complex sampling method aimed to be representative of Brazil. We believe that our results can be generalized to other groups of women. The present study has also some limitations. First of all, the cross-sectional design does not allow us to establish temporal causality. Second, SI was assessed with one single item from the PHQ-9, which was not originally developed to assess the risk of suicide (37). Additional limitations of PHQ-9 item 9 for this type of evaluation are the lack of information about duration and intensity of SI and, more importantly, the difficulty of assessing the person's intention to move from thoughts to action (38). Studies have shown that most patients who answered affirmatively to item 9 did not have imminent risk of suicide (38–40). Authors have also argued that item 9 is ambiguous (41), may elicit different interpretations from different women (42), and is an insufficient assessment tool for suicide risk and suicide ideation, with limited utility in certain demographic and clinical subgroups (43). However, other investigations have considered the PHQ-9 as a robust predictor of suicide attempts and deaths (44). Even though the PHQ-9 is a good screening tool for depression for population survey studies, a better strategy would be a more detailed psychiatric evaluation of depression and suicidality. Another aspect is that the PNS 2019 does not provide information about whom received a diagnosis and how the health care provider gave the participant the diagnosis of depression. The degree of knowledge about depression and other mental health disorders may vary greatly among health care providers. A third limitation may arise in the assessment of SI due to the fact that many women may not feel secure/confident enough to report suicidal ideas (45). Therefore, this information bias could result in a lower prevalence of SI. On the other hand, our assessment of suicidality was limited to the previous 2 weeks (not the previous year or over the lifetime), making recall bias less likely. Fourth, the variable “pregnancy status” was based on the participant's self-report. Unfortunatelly, the PNS 2019 does not provide any confirmation of pregnancy status with sonography or laboratory tests. Fifth, we cannot rule out a certain degree of misclassification regarding our categorization of “recent clinical diagnosis of depression.” However, we believe that if there were women who were depressed and were categorized as “without recent depression” according to our criteria, the effect measure would be greater than the one found in the present study. Therefore, the potential error would be conservative. The assessment of depression would be ideally performed with a psychiatric evaluation with the use of structured diagnostic criteria. Nevertheless, our study is a secondary data analysis from the PNS 2019, a population survey, that used the PHQ-9 to measure depressive symptoms. Finally, recall bias for a clinical diagnosis of depression may have occurred. Depression is often unrecognized by health providers (46) and higher severity of depressive symptoms have been significantly associated with depression detection (47). Furthermore, many women face difficulties in accessing the diagnosis and treatment of depression in health centers. Overall, we may expect in our study that the true prevalence of clinical depression could be higher than the prevalence reported here.

## Conclusion

SI is a common problem for reproductive-aged women and the risk of SI associated with pregnancy varies according to the duration of the clinical diagnosis of depression. Among non-depressed women, pregnant women showed a lower risk of SI in comparison with non-pregnant women. In contrast, in the presence of a recent clinical diagnosis of depression pregnancy increased the risk of SI. Management of SI among pregnant women should correctly identify sociodemographic risk factors and the presence of a recent clinical diagnosis of depression. Efforts to prevent the negative consequences of SI during pregnancy should target high risk of pregnant women diagnosed with depression.

## Data availability statement

Publicly available datasets were analyzed in this study. This data can be found at: https://www.ibge.gov.br/en/statistics/social/justice-and-security/16840-national-survey-of-health.html?edicao=19375.

## Ethics statement

The studies involving human participants were reviewed and approved by the PNS study and the National Research Ethics Committee (Conselho Nacional de Ética em Pesquisa - CONEP). Written informed consent for participation was not required for this study in accordance with the national legislation and the institutional requirements.

## Author contributions

All authors listed have made a substantial, direct, and intellectual contribution to the work and approved it for publication.

## Conflict of interest

The authors declare that the research was conducted in the absence of any commercial or financial relationships that could be construed as a potential conflict of interest.

## Publisher's note

All claims expressed in this article are solely those of the authors and do not necessarily represent those of their affiliated organizations, or those of the publisher, the editors and the reviewers. Any product that may be evaluated in this article, or claim that may be made by its manufacturer, is not guaranteed or endorsed by the publisher.
